# Another New Year, Will the Chinese Residents Wear Face Masks Again? A Cross-Sectional Survey

**DOI:** 10.3389/fpubh.2021.727234

**Published:** 2021-11-10

**Authors:** Xin Shen, Shijiao Yan, Hui Cao, Jing Feng, Zihui Lei, Yuxin Zhao, Zhenyu Nui, Xiaotong Han, Chuanzhu Lv, Yong Gan

**Affiliations:** ^1^Department of Social Medicine and Health Management, School of Public Health, Tongji Medical College, Huazhong University of Science and Technology, Wuhan, China; ^2^School of Public Health, Hainan Medical University, Haikou, China; ^3^Key Laboratory of Emergency and Trauma of Ministry of Education, Hainan Medical University, Haikou, China; ^4^Department of Labor Economics and Management, Beijing Vocational College of Labour and Social Security, Beijing, China; ^5^Community Health Service Management Center, Shenzhen Fuyong People's Hospital, Shenzhen, Guangdong, China; ^6^Department of Public Health and Preventive Medicine, Medical College of Shihezi University, Shihezi, China; ^7^Department of Emergency Medicine, Hunan Provincial Institute of Emergency Medicine, Hunan Provincial Key Laboratory of Emergency and Critical Care Metabolomics, Hunan Provincial People's Hospital/The First Affiliated Hospital, Hunan Normal University, Changsha, China; ^8^Emergency Medicine Center, Sichuan Provincial People's Hospita, University of Electronic Science and Technology of China, Chengdu, China; ^9^Research Unit of Island Emergency Medicine, Chinese Academy of Medical Sciences (No. 2019RU013), Hainan Medical University, Haikou, China

**Keywords:** face masks, low transmission period, interpersonal communication, COVID-19, public health

## Abstract

**Background:** As more and more countries enter the low-transmission phase, maintaining prevention awareness among the population is critical to prevent a secondary outbreak. With large-scale interpersonal communication, whether Chinese residents can maintain a high awareness of prevention and control and adhere to the use of masks during the Chinese New Year of 2021 is worth studying.

**Methods:** A cross-sectional survey was conducted in China from February 4 to 26, 2021. A convenient sampling strategy was adopted to recruit participators. Participants were asked to fill out the questions that assessed the questionnaire on face mask use. Descriptive statistics were used to assess the mask-wearing behaviors of the public. A binary logistic regression analysis was performed to identify the risk factors affecting mask-wearing behaviors.

**Results:** A total of 2,361 residents filled out the questionnaire. In the mixed-effect logistic regression analysis, Chinese residents who were older (OR = 7.899, 95%CI = 4.183–14.916), employed (OR = 1.887, 95%CI = 1.373–2.594), had a chronic disease (OR = 1.777, 95%CI = 1.307–2.418), reused face masks (OR = 22.155, 95%CI = 15.331–32.016) and have read the face mask instructions (OR = 3.552, 95%CI = 1.989–6.341) were more likely to use face masks in interpersonal communication during the Spring Festival; while people who have breathing discomfort caused by face masks (OR = 0.556, 95%CI = 0.312–0.991) and considered that using masks repeatedly is wasteful (OR = 0.657, 95%CI = 0.482–0.895) were more unlikely to use face masks.

**Conclusions:** Our results revealed that 83.86% of people wore face masks during the Chinese New Year; however, some aspects require further promotion. By investigating the use of masks by Chinese residents during the Spring Festival and its influencing factors, we can reflect the prevention awareness of the residents during the low transmission period of COVID-19, which can provide a reference for Chinese and global public health policymakers.

## Background

The origin of the outbreak of COVID-19 was initially detected in Wuhan, China in December 2019 (1). The virus is mainly transmitted from person to person through the mouth, nose, or eyes through respiratory droplets, aerosols, or contaminants (2, 3).

It can live on surfaces for up to 72 h (4) and contact with contaminated surfaces and then touching the face is another possible source of transmission (5). Wearing a face mask is a reasonable way to reduce the spread of respiratory viruses and minimizes the risk of respiratory droplets reaching the nasal or oral mucosa of the wearer (5).

A growing number of places recommend wearing masks in community settings. The WHO and the U.S. Centers for Disease Control and Prevention (6) strongly recommend that people with symptoms or known infections wear masks to prevent the transmission of severe acute respiratory syndrome coronavirus 2 (SARS-CoV-2) to others (7). There is some evidence supporting the effectiveness of using masks in health care settings (8, 9) and as source control for patients infected with SARS-CoV-2 or other coronaviruses (10). Wearing a mask in a community setting is recommended to reduce the transmission of SARS-CoV-2 (7, 9) as it protects the uninfected wearers (protective effect) and reduces the transmission from the infected wearers (source control).

The Chinese New Year holiday, which coincides with the COVID-19 outbreak, is one of the most festive times of the year in China, with a lot of human interaction (11). The Chinese government quickly proclaimed prevention and control measures for interpersonal communication during the Spring Festival, especially the use of face masks, to impede transmission in health care and community settings (12). In the year 2020, the Chinese government strongly advocated the universal use of face masks in public places as a means of source control during the COVID-19 pandemic (13). Chinese residents generally supported the use of masks in public places (14) as a supplement to social distancing and hand hygiene to contain or slow the exponential growth of the epidemic (15). Universal masking prevents the inevitable cross-spread of person-to-person contact during the lockdown and reduces the risk of a resurgence during the relaxation of social distancing measures.

Currently, the COVID-19 epidemic has been controlled and China has entered a period of low transmission (16). At this stage, the Chinese government is still asking the public to increase their vigilance against COVID-19, keeping the use of face masks in communities and reducing concentration. February 4–26, 2021, is considered the Chinese New Year. With the large-scale interpersonal communication, whether or not Chinese residents can maintain a high awareness of prevention and control and adhere to the use of masks is worth studying. As more and more countries enter the low-transmission phase, maintaining prevention awareness among the population is critical in preventing a secondary outbreak. By investigating the use of masks by Chinese residents during the Spring Festival and its influencing factors, we can reflect on the prevention awareness of the residents during the low transmission period of COVID-19, which can provide a reference for the Chinese and global public health policymakers.

## Methods

### Ethics Statement

This study scheme was approved by the Institutional Review Committee of Tongji Medical College, Huazhong University of Science and Technology, Wuhan, China. All the methods are performed in accordance with relevant guidelines and regulations. The respondents were informed that their participation was voluntary and implied consent on the completion of the questionnaire.

### Study Participants and Survey Design

A cross-sectional survey was conducted in China from February 4 to 26, 2021. We stratified the respondents mainly according to the eastern, central, and western regions of China. We selected residents from eastern (Beijing, Tianjin, Hebei, Liaoning, Shanghai, Jiangsu, Zhejiang, Fujian, Shandong, Guangdong, and Hainan), central (Shanxi, Jilin, Heilongjiang, Anhui, Jiangxi, Henan, Hubei, and Hunan), and western (Chongqing, Sichuan, Guizhou, Yunnan, Tibet, Shaanxi, Gansu, Qinghai, Ningxia, Xinjiang, Inner Mongolia, and Guangxi) China to complete the survey. A convenient sampling strategy was adopted to recruit the participants; the research team used WeChat, the most popular social media platform in China, to publicize and distribute the survey links to their network members. The network members were asked to distribute the survey invitations to all of their contacts. The participants were informed that their participation was voluntary and their consent was implied by their completion of the questionnaire. The applicants should be Chinese citizens aged 18 or above and are able to understand and read Chinese.

### Instruments

The questionnaire was compiled according to the guidelines issued by the National Health Commission of China (17, 18). The final version of the questionnaire was entitled “Questionnaire on Face Masks Use for the Public during the 2021 New Year (Except Healthcare Workers)” and consisted of two parts: (1) socio-demographic characteristics, with seven items, including gender, age, highest educational level, place of residence, religion, employment status, and “have a chronic disease (diagnosed by a doctor),” and (2) mask-wearing behaviors and attitudes, with 16 items, including “main types of face masks,” “main sources of face masks,” “reuse face masks,” “have read the face mask instructions,” “face masks cause breathing discomfort,” “consider that using masks repeatedly is wasteful,” “consider that using masks repeatedly is too troublesome,” and “clean the used masks before discarding.”

The electronic (e)-questionnaires were compiled using Wenjuanxing (www.wjx.cn), a survey platform widely used in China. Online posters with access codes or links to the questionnaires were distributed in one of these two ways:(1) posted on our WeChat; (2) distribution was made through the WeChat group, and each person was to be compensated 1–2 ¥ on average. To avoid duplicate submissions, each person can only participate one time for each WeChat account.

### Statistical Methods

The data were analyzed using *SPSS*™ for Windows, Version 22.0 (SPSS, Inc., Chicago, Illinois, United States). We dichotomized the answers of the residents regarding their willingness to use face masks as “Yes” and “No.” The descriptive statistics were presented as the percentage (%) of the number of observations, and we analyzed the differences in the demographic statistics using a Chi-square (χ^2^) test. Due to the disparities in the socioeconomic status in the different regions, the data has a typical hierarchical structure. We performed a mixed-effect logistic regression model with a random cluster effect (geographic regions) to investigate the adjusted odds ratio (OR) (95% CI) of the influencing factors of the willingness of residents to use face masks. Further, we explored the factors influencing the willingness of the participants to use face masks in Eastern, Central, and Western China, respectively, through multivariable logistic regression analysis. The significance level was accepted as *P* < 0.05 (two-sided).

## Results

### Descriptive Statistics

A total of 2,453 residents received the questionnaire, of which 21 did not reply and 71 did not accomplish the questionnaire. The response rate was 96.24%. The results were analyzed using the 2,361 complete questionnaires. Table 1 reports the social-demographic characteristics of the 2,361 respondents. The mean age was 29.72 years (SD = 6.94) and the majority of respondents were female (60.10%). Among the respondents, 421 (17.83%), 1,470 (62.26%), a1nd 470 (19.91%) were from Eastern, Central, and Western China, respectively. Most respondents (89.24%) have a bachelor's degree or higher. More than half of the participants (57.05%) were unemployed.

**Table 1:** The statistical description of the study samples: univariate analysis of the differences of the willingness of residents to use face masks in their interpersonal communication during the Spring Festival.

**Variables**	***N* (%)**	*****χ^2^*****	***P***
Total	2,361 (100)	NA	NA
Using face masks in interpersonal communication during the Spring Festival			
Yes	1,980 (83.86)	NA	NA
No	381 (16.64)		
Gender			
Male	942 (39.90)	4.140	0.042
Female	1,419 (61.10)		
Age group, y			
18–44	1,845 (78.14)	36.610	<0.001
45–59	369 (15.63)		
>60	111 (4.70)		
Highest educational level			
Primary school or below	68 (2.88)	1.738	0.419
Middle school	186 (7.88)		
College degree or above	2,107 (89.24)		
Place of residence			
Urban	1,372 (58.11)	14.202	<0.001
Rural	989 (41.89)		
Region			
Eastern China	421 (17.83)	17.906	<0.001
Central China	1,470 (62.26)		
Western China	470 (19.91)		
Employment status			
Employed	1,014 (42.95)	21.262	<0.001
Unemployed	1,347 (57.05)		
Have a chronic disease (diagnosed by a doctor)			
Yes	1,621 (68.66)	226.324	<0.001
No	740 (31.34)		
Main types of face masks			
Respirators (N95 and FFP)	614 (26.01)	3.343	0.188
Surgical mask	1,059 (44.85)		
Cloth masks	688 (29.14)		
Main sources of face masks			
Purchased	2,023 (85.68)	86.693	<0.001
Free (community, work unit, etc provide)	338 (14.32)		
Reuse face masks			
Yes	1,435 (60.78)	1,279.010	<0.001
No	926 (39.22)		
Have read the face mask instructions			
Yes	1,136 (48.12)	831.344	<0.001
No	1,225 (51.88)		
Face masks cause breathing discomfort			
Yes	1,224 (52.69)	781.840	<0.001
No	1,117 (47.31)		
Consider that using masks repeatedly is wasteful			
Yes	726 (30.75)	181.024	<0.001
No	1,635 (69.25)		
Consider that using masks repeatedly is too troublesome			
Yes	622 (26.34)	0.905	0.341
No	1,739 (73.66)		
Clean the used masks before discarding			
Yes	239 (10.12)	0.023	0.880
No	2,122 (89.88)		

Out of all the participants, 1,980 (83.86%) have used face masks in their interpersonal communication during the Spring Festival. The results of the univariate analysis suggested that the gender, age, place of residence, region, employment status, “have a chronic disease,” “main sources of face masks,” “reuse face masks,” “have read the face mask instructions,” “face masks cause breathing discomfort,” and “consider that using masks repeatedly is wasteful” were statistically significant influencing factors for “using face masks in interpersonal communication during the Spring Festival”(*P* < 0.05) (Table 1).

Univariate analysis of the participants from Eastern, Central, and Western China was conducted, taking the sampling differences across the geographical regions into account (Table 2). In the mixed-effect logistic regression analysis, the Chinese residents who were older (OR = 7.899, 95%CI = 4.183–14.916), employed (OR = 1.887, 95%CI = 1.373–2.594), had a chronic disease (OR = 1.777, 95%CI = 1.307–2.418), reused face masks (OR = 22.155, 95%CI = 15.331–32.016), and have read the face mask instructions (OR = 3.552, 95%CI = 1.989–6.341) were more likely to use face masks in their interpersonal communication during the Spring Festival, while the people who experienced breathing discomfort from face masks (OR = 0.556, 95%CI = 0.312–0.991) and considered that using masks repeatedly is wasteful (OR = 0.657, 95%CI = 0.482–0.895) were not inclined to use face masks (Table 3).

**Table 2:** Univariate analysis of the differences in the willingness to use face masks in interpersonal communication during the Spring Festival among the included residents stratified by geographic characteristics.

**Variables**	**Eastern China**			**Central China**			**Western China**		
	***N* (%)**	*****χ^2^*****	***P***	**N (%)**	*****χ^2^*****	***P***	**N (%)**	*****χ^2^*****	***P***
Total	421 (100)	NA	NA	1,470 (100)	NA	NA	470 (100)	NA	NA
Using face masks in interpersonal communication during the Spring Festival									
Yes	367 (87.17)	NA	NA	1,185 (80.61)	NA	NA	428 (91.06)	NA	NA
No	54 (12.83)			285 (19.39)			42 (8.94)		
Gender									
Male	181 (42.99)	0.162	0.687	558 (37.96)	1.675	0.196	203 (43.19)	2.353	0.125
Female	240 (57.10)			912 (62.04)			267 (56.81)		
Age group, y									
18–44	323 (76.72)	7.089	0.029	1,175 (79.93)	19.178	<0.001	347 (73.83)	7.648	0.022
45–59	61 (14.49)			232 (15.78)			76 (16.17)		
>60	37 (8.79)			63 (4.29)			47 (10.00)		
Highest educational level									
Primary school or below	17 (4.04)	2.454	0.293	27 (1.84)	2.519	0.284	24 (5.11)	2.092	0.351
Middle school	24 (5.70)			123 (8.37)			39 (8.30)		
College degree or above	38 (90.26)			1,320 (89.80)			407 (86.60)		
Place of residence									
Urban	288 (68.41)	0.442	0.506	754 (51.29)	8.185	<0.001	330 (70.21)	1.624	0.203
Rural	133 (31.59)			716 (48.71)			140 (29.79)		
Employment status									
Employed	270 (64.13)	0.091	0.763	508 (34.56)	35.699	<0.001	236 (50.21)	3.508	0.061
Unemployed	151 (35.87)			962 (65.44)			234 (49.79)		
Have a chronic disease (diagnosed by a doctor)									
Yes	286 (67.93)	40.954	<0.001	985 (67.01)	140.892	<0.001	350 (74.47)	38.785	<0.001
No	135 (32.07)			485 (32.99)			120 (25.53)		
Main types of face masks									
Respirators (N95 and FFP)	97 (23.04)	5.342	0.069	405 (27.55)	2.364	0.307	112 (23.83)	1.388	0.500
Surgical mask	200 (40.51)			637 (43.33)			222 (47.23)		
Cloth masks	124 (29.45)			428 (29.12)			136 (28.94)		
Main sources of face masks									
Purchased	357 (84.80)	10.902	<0.001	1,259 (85.65)	58.561	<0.001	407 (86.60)	17.841	<0.001
Free (community, work unit, etc provide)	64 (15.20)			211 (14.35)			63 (13.40)		
Reuse face masks									
Yes	259 (61.52)	169.104	<0.001	854 (58.10)	863.413	<0.001	322 (68.51)	241.527	<0.001
No	162 (38.48)			616 (41.90)			148 (31.49)		
Have read the face mask instructions									
Yes	199 (47.27)	132.368	<0.001	672 (45.71)	543.494	<0.001	265 (56.38)	148.066	<0.001
No	222 (52.73)			798 (54.29)			205 (43.62)		
Face masks cause breathing discomfort									
Yes	212 (20.36)	122.873	<0.001	815 (55.44)	520.793	<0.001	217 (46.17)	131.530	<0.001
No	209 (49.64)			655 (44.56)			253 (53.83)		
Consider that using masks repeatedly is wasteful									
Yes	139 (33.02)	34.222	<0.001	462 (31.43)	112.694	<0.001	125 (26.60)	31.417	<0.001
No	282 (66.98)			1,008 (68.57)			345 (73.40)		
Consider that using masks repeatedly is too troublesome									
Yes	109 (25.89)	0.159	0.690	393 (26.73)	1.055	0.304	120 (25.53)	0.342	0.559
No	312 (74.11)			1077 (73.27)			350 (74.47)		
Clean the used masks before discarding									
Yes	37 (8.79)	0.920	0.337	151 (10.27)	1.129	0.288	51 (10.85)	0.689	0.407
No	384 (91.21)			1,319 (89.73)			419 (89.15)		

**Table 3:** Mixed-effect logistic regression analysis on the influencing factors of the willingness of residents to use face masks in interpersonal communication during the Spring Festival.

**Variables**	**Coefficient**	***S.E*.**	***P***	***OR***	**95% *CI***
Age group, y (Ref: 18–44)					
45–59	0.597	0.191	0.002	1.817	1.250–2.641
>60	2.067	0.324	<0.001	7.899	4.183–14.916
Employment status (Ref: Unemployed)					
Employed	0.635	0.162	<0.001	1.887	1.373–2.594
Have a chronic disease (Ref: No)					
Yes	0.575	0.157	<0.001	1.777	1.307–2.418
Reuse face masks (Ref: No)					
Yes	3.098	0.188	<0.001	22.155	15.331–32.016
Have read the face mask instructions (Ref: No)					
Yes	1.267	0.296	<0.001	3.552	1.989–6.341
Face masks cause breathing discomfort (Ref: No)					
Yes	−0.587	0.295	0.046	0.556	0.312–0.991
Consider that using masks repeatedly is wasteful (Ref: No)					
Yes	−0.420	0.158	0.008	0.657	0.482–0.895

In addition, we stratified the study samples by region and performed multivariate logistic regression analysis. The results showed that the “main source of purchase of face masks” (OR = 32.587, 95%CI = 19.439–54.629) was also a related factor for the increase in the willingness to use face masks among residents in Central China (Table 4).

**Table 4:** Stepwise multivariate logistic regression analysis on the influencing factors of the willingness of residents to use face masks in interpersonal communication during the Spring Festival.

**Variables**	**Coefficient**	***S.E*.**	***P***	***OR***	**95% *CI***
**Eastern China**					
Age group, y (Ref: 18–44)					
>60	1.945	0.602	0.001	6.993	2.149–22.757
Have a chronic disease (Ref: No)					
Yes	0.698	0.354	0.049	2.010	1.004–4.024
Reuse face masks (Ref: No)					
Yes	2.257	0.391	<0.001	22.155	15.331–32.016
Have read the face mask instructions (Ref: No)					
Yes	2.290	0.638	<0.001	9.878	2.826–34.528
Consider that using masks repeatedly is wasteful (Ref: No)					
Yes	−0.420	0.158	0.008	0.657	0.482–0.895
**Central China**					
Age group, y (Ref: 18–44)					
>60	1.776	0.520	0.001	5.908	2.131–16.377
Employment status (Ref: Unemployed)					
Employed	1.155	0.219	<0.001	3.175	2.067–4.876
Have a chronic disease (Ref: No)					
Yes	0.691	0.206	0.001	1.997	1.334–2.988
Main sources of face masks (Ref: Free)					
Purchased	0.524	0.245	0.032	1.689	1.045–2.730
Reuse face masks (Ref: No)					
Yes	3.484	0.264	<0.001	32.587	19.439–54.629
Have read the face mask instructions (Ref: No)					
Yes	1.035	0.395	0.009	2.816	1.298–6.109
Consider that using masks repeatedly is wasteful (Ref: No)					
Yes	−0.458	0.207	0.027	0.633	0.421–0.949
**Western China**					
Age group, y (Ref: 18–44)					
45–59	1.345	0.458	0.003	3.838	1.565–9.412
>60	1.790	0.656	0.006	5.992	1.658–21.660
Reuse face masks (Ref: No)					
Yes	3.777	0.508	<0.001	43.699	16.134–118.355

## Discussion

Our study, based on a cross-sectional survey, determined the willingness to use face masks in interpersonal communication and its influencing factors during the Spring Festival among Chinese residents. We found that 83.86% of the citizens have used the face masks, and this rate is lower than that in another study about the rate of face masks usage among Chinese citizens (99%) by Tan et al. (14) during the rapid spread of COVID-19. Nearly one-fifth of the participants demonstrated bad compliance in terms of mask-wearing behaviors in the period of low transmission without realizing the risk of intense interpersonal communication. Moreover, this study found some factors associated with good compliance, including age, employment status, “have a chronic disease,” “reuse face masks,” “have read the face mask instructions,” “face masks cause breathing discomfort,” “consider that using masks repeatedly is wasteful,” and “main sources of face masks.”

Age can be a factor in mask-wearing behavior. Consistent with previous studies that examined the changes in public behavior during influenza outbreaks (19, 20), the older participants in our survey showed a trend toward better compliance with age. In addition, the people who were unemployed exhibited better compliance with face mask use than the employed participants. This phenomenon may be related to the lower risk resistance and psychological resilience of the elderly and the non-employed (21). In addition, as a survey method of convenience sampling was adopted in this study, many students were included as participants. Therefore, the proportion of unemployed respondents is relatively large. The results of this analysis should be treated with more caution.

We also observed that different situations affect the behavior of people. Compliance is much better in patients with chronic diseases. This may be due to their concerns about the high risk of COVID-19 transmission in these settings and the association between the perception of high risk and good compliance with mask use (19, 22). Similarly, compliance was worse among residents who agreed that wearing masks caused discomfort in breathing. In real life, when people have symptoms of breathing disorders, they may feel uncomfortable, and the frequent use of masks can lead to worse compliance (14).

Among the factors influencing mask-wearing behaviors, we found that people exposed to the instructions on how to use masks showed better compliance than people who are not. Interestingly, there was no significant relationship between educational background and compliance. Thus, good mask-wearing habits seem to depend on how much education is received about mask use, rather than on the level of education. This finding also supported the hypothesis proposed by Greenhalgh et al. (23) that, in the case of COVID-19, people can be taught to use masks properly and to stick with them without abandoning other important anti-infection measures. This evidence, combined with our findings on the methods by which the participants obtained relevant information, suggests that institutions and the academe should put effort into dissemination guidance through a variety of means, of which social media is the most beneficial to the public.

Economic factors are crucial. The participants who considered that using masks repeatedly is wasteful are more likely to refuse using face masks. Similarly, reusing face masks also increases compliance. Whether or not people will reduce their health protection to save money has not been reported, but it is still an important Research Topic during periods of low transmission. Notably, the residents who bought masks out of their own pocket also demonstrated low compliance, but this phenomenon was only seen in central China, thus the effect of this factor needs further investigation.

### Strengths and Limitations

Wearing masks in large-scale interpersonal interactions can reflect the awareness of residents regarding the prevention of COVID-19. This is the first study to investigate the mask-wearing behaviors of the general public in the period of low COVID-19 transmission. We used a nationwide sample of the Chinese population. The findings provide evidence about the way the public uses masks and the factors that influence their behavior, which is of great significance to China and other countries. First of all, this research takes social media as the main communication survey method. Participants who do not have Internet access may not be included. Therefore, the strategy of simultaneous online and offline development should be adopted in future research. Online surveys rely on social software, while offline surveys rely on community or rural health service institutions, medical personnel, and primary management personnel. This method of the survey will include a wider range of residents and reduce the bias caused by online surveys. Second, the study participants were unevenly distributed in different regions (421:1,470:470). Therefore, the subgroups of the variables may not be representative of the population. Third, the study was unable to determine how many participants have seen the online posters or surveys but decided not to complete them, therefore, the existence of the non-response bias cannot be evaluated. Finally, since these behaviors are self-reported, reporting bias is possible. In general, the generalization of the results should be viewed with caution.

## Conclusions

Because of the highly infectious nature of COVID-19 and the ongoing severity of the global epidemic, wearing masks has become a part of daily life. Although more and more countries are entering the low-infection period, face masks can still play an important role in preventing a second outbreak. Therefore, understanding how the public uses masks and what factors are associated with good compliance will help determine ways to promote proper mask-wearing behaviors.

Our results show that 83.86% of the Chinese residents wore masks during the Spring Festival. However, there are still some areas that need further promotion. In future evidence dissemination or behavior change interventions, particular emphasis should be placed on wearing masks among young people, employed persons, and healthy residents. In addition, the reusing of masks and the instructions for the use of masks should not be ignored. In the period of low transmission, it is important to take as many publicity measures as possible to promote the wearing of masks by the public. Therefore, different influencing factors should be considered in the dissemination of evidence to reach different populations. Methods should be adopted for the clear and ubiquitous dissemination of government warnings and alerts. Social media is the most powerful way to reach an audience and facilitate data collection. However, further research on how social media can promote public behavior change is needed.

## Data Availability Statement

The raw data supporting the conclusions of this article will be made available by the authors, without undue reservation.

## Ethics Statement

This study protocol was approved by the Institutional Review Board of the Tongji Medical College of Huazhong University of Science and Technology, Wuhan, China.

## Author Contributions

XS, SY, and YG conceived and designed the study. JF and ZL participated in the acquisition of data. XS and SY analyzed the data and drafted the manuscript. HC, YZ, and ZN provided advice for the methodology. YG, CL, and XH revised the manuscript. YG is the guarantor of this work and had full access to all the data in the study and takes responsibility for its integrity and the accuracy of the data analysis. All authors contributed to the article and approved the submitted version.

## Funding

This work was supported by the Fundamental Research Funds for the Central Universities (2020kfyXJJS059).

## Conflict of Interest

The authors declare that the research was conducted in the absence of any commercial or financial relationships that could be construed as a potential conflict of interest.

## Publisher's Note

All claims expressed in this article are solely those of the authors and do not necessarily represent those of their affiliated organizations, or those of the publisher, the editors and the reviewers. Any product that may be evaluated in this article, or claim that may be made by its manufacturer, is not guaranteed or endorsed by the publisher.
